# Human stem cell-based embryo models and the 2025 ISSCR Guidelines

**DOI:** 10.1186/s13287-026-05179-y

**Published:** 2026-07-29

**Authors:** Jonathan Lewis, Søren Holm

**Affiliations:** 1 School of Law, Faculty of Arts, Humanities, and Social Sciences, Ulster University, Belfast, UK; 2 School of Medicine, Faculty of Life and Health Sciences, Ulster University, Derry/Londonderry, UK; 3 Present Address: Centre for Social Ethics and Policy, Department of Law, School of Social Sciences, The University of Manchester, Oxford Road, Manchester, M13 9PL UK; 4 Center for Medical Ethics, HELSAM, Faculty of Medicine, University of Oslo, Oslo, Norway

## Abstract

With the 2025 revisions to the *ISSCR Guidelines for Stem Cell Research and Clinical Translation*, human stem cell-based embryo models (SCBEMs) sit within a clearer governance framework. We argue, however, that four practical challenges require further clarification. First, specialized oversight of human SCBEM research could be delayed where integration-building work is distributed across several protocols. Second, ambiguity persists at the boundary between SCBEMs and stage- or structure-specific 3D constructs. Third, the new oversight standards for human SCBEM research are not explicitly linked to the ethical principles set out in the *Guidelines*. Finally, animal implantation or gestation findings could be accorded too much weight when committees consider the limits and scope of human SCBEM safeguards. In light of these four challenges, we propose four targeted clarifications: (1) a narrowly framed *consultation trigger* for defined protocol-level integration-building manipulations, without moving ordinary organoid, endometrial, placental, or trophoblast research into Category 2; (2) a Category 3B clarification distinguishing permissible, time-limited implantation modelling from impermissible attempts to enable sustained gestational or ectogenetic progression; (3) guidance limiting the *decisive* inferential use of animal SCBEM gestation evidence in human SCBEM oversight; and (4) a short note linking SCBEM classification and the ISSCR’s ethically motivated limits on preclinical human SCBEM research to explicit criteria and ethical justification.

## Introduction

The International Society for Stem Cell Research (‘ISSCR’) defines its relevant categories of ethical review as follows:

- Category 1 A: Exempt from review by a specialized oversight process.
- Category 1B: Reportable, but not typically reviewed by a specialized oversight process.
- Category 2: Reviewed by a specialized oversight process.
- Category 3 A: Not allowed; currently unsafe.
- Category 3B: Not allowed: lacks compelling scientific rationale or is ethically concerning.

The 2025 update to the *ISSCR Guidelines for Stem Cell Research and Clinical Translation*—following, in large part, the recommendations of the ISSCR’s working group (see [3])—shifts oversight of human SCBEM research from the previous “integrated vs. non-integrated” distinction to an approach in which every protocol leading to a spatially organized 3D human SCBEM is reviewed under Category 2 ([10], § 2.2.2(g).

Researchers must justify the use of a particular model over simpler alternatives and set explicit culture windows tied to developmental milestones, with extensions requiring re-review (ibid., Recommendation 2.2.2.2). In addition, the revised guidelines prohibit reproductive pathways, with transfers of any human SCBEM to a human or animal uterus and the culture of human SCBEMs in artificial, viability-enabling systems (i.e., ectogenesis) subsumed under Category 3B (ibid., § 2.2.3B(a). Furthermore, the revised guidelines recommend that researchers provide regulatory compliance and ISSCR-category statements in human SCBEM-related research proposals, publications, and preprints [3].

In what follows, we identify and seek to address four challenges facing the revised ISSCR guidelines. First, although § 2.2.2(g) captures ‘all approaches that lead to spatially organized 3D SCBEMs’, ethics review and specialized oversight are normally protocol- or study-specific. Category 2 engagement could, therefore, be delayed where a research programme develops a human SCBEM across separate modules, with each protocol materially increasing the developmental integration of a downstream construct but no individual protocol obviously ‘leading to’ a spatially organized 3D SCBEM. Our concern is not ordinary organoid development or general method-building, but planned integration-building steps involving a human SCBEM or a construct intended, or reasonably expected, to become one.

Secondly, in a previous article, we argued that inferences concerning the developmental potential of human SCBEMs from animal SCBEM implantation or gestation data rest on epistemically tenuous assumptions [13]. The revised guidelines prohibit ectogenesis and the transfer of human SCBEMs to any human or animal uterus, but they do not address how animal SCBEM implantation or gestation data should be used in human SCBEM review. Given species-specific developmental trajectories, and the fact that human-specific in vitro protocols may diverge from what animal systems predict, we caution against placing *decisive* weight on animal SCBEM implantation outcomes when judging what should be permitted in human SCBEM research.

The third challenge concerns the question of what does (and does not) count as an ‘SCBEM’ and, thus, how the ISSCR’s definition of an SCBEM should be operationalized by researchers and oversight committees. We show that definitional ambiguity risks yielding divergent review outcomes, particularly for lineage-incomplete or stage-limited 3D constructs that nevertheless display embryo-level organization or milestones.

The practical import of these challenges can be illustrated by three examples. First, a trophoblast organoid remains Category 1 A when studied on its own, but a protocol proposing to incorporate a trophoblast-like compartment into a pluripotent-cell-derived embryo model construct to generate embryo-level organization should at least trigger consultation with the oversight committee. Second, an endometrial model used to study implantation failure is not itself a SCBEM and should not be moved into Category 2, but co-culture of that model with a human SCBEM falls within the Category 2 framework and requires clear time limits and justification. Third, a lineage-incomplete 3D construct that forms anterior-posterior patterning or primitive-streak-like events may not resemble a whole embryo, but it can still raise ethical concerns that Category 2 is intended to capture.

In terms of a fourth challenge, neither the guidelines nor the preceding article by the ISSCR’s working group (see [3]) explain how certain ethical constraints on human SCBEM research, such as time-limited culture and prohibitions on open-ended culture, relate to the ethical principles outlined in Sect. 1 of the *Guidelines*. More generally, there remains a need for explicit ethical justification for what ultimately amount to ethically motivated restrictions on human SCBEM research.

To address these four challenges, we argue for four targeted clarifications to the updated ISSCR guidelines. These clarifications would: (1) introduce a narrowly framed *consultation trigger* so that defined integration-building manipulations are brought to the attention of the oversight committee at the point they are planned or undertaken, without sweeping ordinary organoid, endometrial, placental, or trophoblast research into Category 2 review; (2) clarify that Category 3B does *not* prohibit time-limited in vitro implantation modelling as such, but, instead, covers protocols whose primary aim is to enable sustained gestational or ectogenetic progression; (3) state that animal SCBEM implantation findings may inform scientific context but should not be treated as *sufficient* or *decisive* grounds to loosen human SCBEM safeguards; and (4) reduce ambiguity by linking SCBEM classification to embryo-level organization or milestones while making explicit the ethical rationale for the ISSCR’s stated limits in preclinical human SCBEM research.

## Clarifying when consultation is needed for integration-building work

By triggering Category 2 review early and broadly—that is, for any approach that ‘leads to’ a spatially organized 3D human SCBEM—the 2025 revisions substantially reduce the risk that ethically salient constructs will escape specialist oversight. They also make it clear that the absence of trophoblast or other extraembryonic cell types is not a criterion for exclusion from review. These changes make the revised scheme less susceptible to inadvertent delay in review via piecemeal assembly, including through integration-building interventions such as trophoblast replacement, which we discussed in our earlier work [13].

Nevertheless, an *implementation gap* remains. Although § 2.2.2(g) captures ‘all approaches that lead to spatially organized 3D SCBEMs’, what counts as ‘leads to’ is often interpreted per protocol rather than across an entire research programme. Laboratories may pursue modules, such as extraembryonic, endometrial, placental, or stage-specific systems in Category 1 A, before any aggregation, assembly, or replacement step clearly yields a spatially organized 3D SCBEM. The ethically salient issue is not the development of these systems in isolation, but the point at which they are deliberately combined with pluripotent-cell derivatives or with a human SCBEM to generate embryo-level organization.

To address this gap without expanding Category 2 in an unmanageable way, we propose a narrowly framed *consultation trigger* within § 2.2.2. This would require investigators to seek oversight committee input, or to file a brief notification, when a specific protocol includes pre-specified manipulations intended, or reasonably expected, to materially increase embryo-level integration in a downstream 3D human SCBEM. The trigger is not aimed at ordinary organoid development, endometrial modelling, placental modelling, trophoblast organoid research, or general method-building, and it should not convert Category 1 A work into full Category 2 review merely because such work could hypothetically be repurposed in the future. Rather, it is designed to capture a defined class of integration-building steps that involve planned assembly, reconstitution, replacement, or co-culture with a human SCBEM or with a construct intended, or reasonably expected, to become a human SCBEM.

For example, a protocol to optimize a trophoblast organoid, endometrial organoid, placental interface, or 3D uterine model would not—in itself—require Category 2 review. Consultation would be appropriate, however, where a protocol proposes to incorporate a trophoblast-like compartment into a pluripotent-cell-derived embryo model to produce coordinated embryo-like patterning, or where an engineered endometrial interface is expressly designed, or reasonably expected, to support implantation-like interaction or sustained post-implantation development of a human SCBEM. In heavily burdened settings, the proposed consultation trigger could operate as a triage device: a brief written justification followed by an oversight committee determination of whether full Category 2 review is warranted.

This clarification is of practical importance because recent in vitro implantation models have demonstrated the potential value of adjacent endometrial, placental, and implantation research. Li and colleagues [16], for example, have recently developed a 3D on-chip implantation model using human blastoids/blastocysts and endometrioids to study implantation, early post-implantation development, and recurrent implantation failure. In addition, Molè and colleagues [18] established a receptive endometrial model in which human embryos and blastoids implant and display post-implantation hallmarks, including trophoblast structures relevant to early placentation. These studies illustrate the scientific value of time-limited implantation modelling and help sharpen the distinction between *permissible* modelling of implantation and ethically *impermissible* attempts to initiate or sustain a *reproductive trajectory*.

Category 3B of the ISSCR guidelines is framed around systems ‘designed to develop SCBEMs to viability’ ([10], § 2.2.3B(a)). As the previous paragraph implies, we are not proposing that in vitro implantation modelling should be prohibited under Category 3B. Time-limited models of apposition, attachment, invasion, trophoblast differentiation, and embryo-endometrial signaling can be scientifically valuable and ethically permissible under Category 2 where the model, time limits, and scientific rationale are clearly justified. Rather, our concern is that the ethical rationale for Category 3B—i.e., to prevent reproductive trajectories that are widely regarded as being unethical—can be undermined if research is permitted when it is oriented toward viability-enabling development. In other words, the relevant ethical distinction is not between implantation research and non-implantation research, but between *time-limited implantation modelling* and attempts to initiate or support a *reproductive or viability-directed trajectory*.

In light of the potential value of model-based, in vitro implantation research (see, e.g., [16, 18]), a modest way to address this challenge would be to protect legitimate implantation modelling while maintaining the prohibition on reproductive trajectories. To this end, we propose the inclusion of *purpose-based clause* to Category 3B targeting protocols whose function or purpose is to enable sustained ectogenetic or gestational progression of a human SCBEM beyond approved time-limited implantation or early post-implantation modelling, including through artificial placental or uterine support systems.

## Using animal evidence cautiously, not decisively

Previous proposals for regulating human SCBEM research have considered whether developmental potential might be assessed by testing the capacity of non-human SCBEMs, for example pig or primate SCBEMs, to form live, fertile animals (see, e.g., [22, 6]). We have argued elsewhere that inferences about the developmental potential of human SCBEMs from non-human implantation or birth studies are epistemically limited [13], since early embryo development, implantation, placentation, birth viability, and epigenetic resetting can vary between species (see, e.g., Biondic et al. [1], Godeau, [8], Hanna, [9], Lee, [11], Liao, [17], Molè, [18], Ogura, [19], Siriwardena, [24]).

Unlike proposals that use tests for developmental potential to justify upper limits to human SCBEM research (see, e.g., [22, 6]), the revised ISSCR guidelines do not rely on live-birth proxy tests to trigger oversight of human SCBEMs. This is a welcome feature of the revised scheme. Nevertheless, animal SCBEM implantation and/or gestation studies may still be invoked in discretionary review contexts when committees consider whether to extend culture windows or permit more developmentally integrated human SCBEM models. The historical fact that non-human models have often paved the way for advances in human stem cell and embryo research supports the *relevance* of animal evidence, but not its *decisiveness* in regulatory contexts. Animal studies can help identify risks, generate hypotheses, and provide relevant biological context. This is an empirical matter of fact, and one on which human stem cell research and the biomedical sciences in general have relied. But whether non-human analogue studies *should* determine the boundaries and scope of human stem cell research is, ultimately, a *normative* question, the answer to which requires ethical reasons developed in light of scientific facts rather than mere appeal to facts themselves. The point is that species-specific differences in implantation, placentation, early development, and epigenetic regulation (see, e.g., Biondic et al., [1], Godeau, [8], Hanna, [9], Lee, [11], Liao, [17], Molè, [18], Ogura, [19], Siriwardena, [24]) undermine whatever normative reasons we might have for treating animal SCBEM implantation or gestation data as *sufficient* or *decisive* grounds for extending human SCBEM culture windows or permitting greater developmental integration (see also [25]). Such decisions require human-specific in-vitro evidence (see [2, 16]) in conjunction with explicit ethical justification (see [13]).

To mitigate this risk, we propose a constraint on the inferential use of animal SCBEM implantation and gestation results in human SCBEM oversight. The aim is not to prevent committees from considering relevant evidence or scientific context. Rather, the guidelines could clarify that animal gestation or implantation outcomes should not be treated as *sufficient* or *decisive* grounds to extend human SCBEM culture limits, relax safeguards, or justify greater developmental complexity in human SCBEMs. Oversight committees should instead ask whether the proposed human SCBEM work is justified by appeal to ethical reasons and human-specific in vitro evidence, including lineage identity, spatial organization, developmental milestones, trophoblast/endometrial interactions, transcriptomic correspondence to human embryo datasets, and evidence of divergence from human embryogenesis. This would preserve scientific responsiveness while guarding against overconfident extrapolation from species-specific developmental trajectories.

Recent human implantation models reinforce this point. Molè and colleagues [18] note that implantation strategies differ between humans and other mammalian species, including primates, in ways that hinder the applicability of non-human systems to human implantation. This supports our limited claim: animal data should remain available to oversight committees, but should not by itself move the ethical goalposts for human SCBEM research.

## Operationalizing the SCBEM boundary

Compared to the previous iteration, the revised ISSCR guidelines tighten the meaning of a SCBEM by supplying a glossary definition and by broadening Category 2 oversight to cover ‘approaches that lead to spatially organized 3D SCBEMs’, explicitly noting that the absence of trophoblast or other extraembryonic tissues is not a basis for exclusion from review ([10], § 2.2.2(g)). At the same time, Category 1 A preserves a carve-out for 3D stem cell-based systems that ‘model specific stages of development or specific anatomic structures and not the continuous development of a 3D spatially organized SCBEM’ (ibid., § 2.2.1 A(e-f)).

The ambiguity between what should fall under Category 1 A and Category 2 is easiest to see by contrasting clear and borderline cases. A trophoblast organoid or yolk-sac organoid remains Category 1 A because it models an extraembryonic structure rather than embryo-level organization or continuous embryo-like development. A blastoid, by contrast, clearly falls within Category 2 because it is a spatially organized 3D construct designed to model key features of the blastocyst. The harder cases are partial or lineage-incomplete 3D constructs that nevertheless display embryo-level axes or milestones: for example, gastruloids, somitoids, axioloids, neuruloids, anterior-posterior organoids, PASE, peri-gastruloids, human embryonic disc-like structures, and structured assembloids. Clark and colleagues [3] treat such systems as Category 2 exemplars, but the *Guidelines* themselves do not reproduce an operational list or provide criteria explaining when a stage-specific model has crossed into Category 2, embryo-level organization.

We, therefore, propose not a new category, but an interpretive note. A 3D construct should be treated as a SCBEM for Category 2 purposes where it includes pluripotent stem cell derivatives and is designed to model embryo-level organization or milestones in 3D, even if the construct is lineage-incomplete *or* temporally limited in its capacity for continuous development. Conversely, structures lacking pluripotent tissue derivatives and aimed solely at modelling extraembryonic tissues should remain Category 1 A. This note would translate the examples provided by Clark and colleagues [3] into action-guiding criteria while preserving the structure and spirit of the revised *Guidelines*.

## Making explicit the ethical rationale for SCBEM-specific standards

Ethics is a normative discipline, and, broadly speaking, this means that it has two primary functions: (1) to provide action guidance; (2) to provide action justification. While there are different views about how and in what form ethical content should be imported into regulatory documents such as the ISSCR’s guidelines, it’s reasonable to assume that, in virtue of being forms of ethics, such guidelines should share these functions and aim to provide guidance and justification for action in biomedical research, clinical translation, and healthcare in general [14, 15].

The ISSCR guidelines are better than many other ethical guidelines in acknowledging the importance of grounding the document in ethical content. Section 1 (‘Fundamental Ethical Principles’) recognizes that the success of the biomedical research landscape—and, by implication, the successful uptake of the guidelines themselves—depends on how research and its associated scientific and ethical standards are perceived. The ISSCR frames this in terms of the importance of supporting and promoting the confidence and trust of a broad range of stakeholders, including patients, healthcare practitioners, funders, and the public in general. At the same time, the ISSCR acknowledges that the ethical demands of the guidelines need to be practically workable at the level of research and clinical translation. In particular, ethical requirements should not unduly undermine or limit the sustainability of biomedical research or the levels of certainty required by those who, from a resource perspective, facilitate and support research (see, for example, [12]).

Many ethical guidance documents present their substantive ethical content in the form of *principles*, i.e., general norms of conduct that describe obligations, permissible and impermissible actions, and/or various ideals of action. And the ISSCR guidelines are no exception, with its principles of ‘integrity of the research enterprise’, ‘primacy of patient/participant welfare’, transparency’, and ‘social and distributive justice’.

There is a long-standing bioethical debate about whether such a *principlist* approach can deliver adequate action guidance or action justification (for overviews, see [15, 14]). Putting that debate aside, it has been argued that the successful uptake of principle-based guidelines requires explicit ‘reasons and reasoning that those affected [by the guidelines’ recommendations] can perceive as legitimate even if they do not endorse the recommendations themselves’ ([14], p. 144). In other words, in order to fulfil its stated mission for biomedical research, the ISSCR should take seriously the requirements for publicly transparent justifications for its recommendations as well as the need for transparent and consistent decision-making in light of those recommendations. Otherwise, there is a risk that the named principles will ‘primarily function as checklists, naming issues worth remembering when one is considering a biomedical moral issue. “Consider this … consider that … remember to look for …’ is what they tell the agent; they [will] not embody an articulated, established, and unified moral system capable of providing guidance’ ([7], p. 75).

There is an open question of whether and to what extent the principles expressed in Sect.  1 of the *Guidelines* provide adequate action guidance and/or justification for all of the ISSCR’s recommendations and standards. This question is beyond the scope of this paper to answer in general, but it is worth noting that the principles seem most directly action-guiding in relation to clinical translation and human subjects research. By contrast, several recommendations concerning human SCBEMs appear to be motivated by specifically ethical considerations: i.e., justifications related to the complexity of a particular model, milestone-tied time limits on model culture, prohibitions on open-ended culture, compliance/category statements in publications, and the Category 3B prohibition on reproductive trajectories. Although the ISSCR supports Category 3B prohibitions by reference to broad international consensus that such experiments lack a compelling scientific rationale or are widely considered unethical, there is little in Sect.  1 of the *Guidelines* that directly explains why these specific Category 2 constraints follow from the stated principles. Without explicit ethical reasons for why developmental culture of SCBEMs should be time-limited, why open-ended culture should be prohibited, or why particular developmental milestones matter, there is a risk of inconsistent decision-making and avoidable uncertainty for researchers, funders, and oversight committees.

To address this gap, we propose that the ethical justification for SCBEM-specific constraints should be made explicit. The *Guidelines* could either include a short SCBEM-specific subsection under Sect. 1 or cross-reference a new note in § 2.2.2 spelling out the rationale for complexity-tied justifications, time-limited culture, and prohibitions on open-ended culture in preclinical SCBEM research. For example, such justifications might appeal to: (1) a principled precautionary stance about uncertain developmental capacities; (2) respect for reasonable pluralism about the moral status of the embryo; (3) concern about non-instrumentalizable thresholds; and/or (4) the need to sustain public trust in a rapidly developing and ethically sensitive research field (for details of how the ISSCR might justify its ethical recommendations for human SCBEM research, see, for example, [4, 5, 13, 20, 23]). Providing these sorts of reasons would link the language of the ISSCR’s stated ethical mission to the concrete, action-guiding constraints on human SCBEM research and would equip committees to defend stable limits against shifting empirical claims.

This concern is not merely about whether the resulting rules are substantively defensible. It is also about how ethical legitimacy is generated. The involvement of leading SCBEM scientists in guideline development is both inevitable and valuable, since the relevant scientific distinctions, concepts, and state of the art are technically complex. However, where ethical guidance is developed by experts who may also be members of the regulated community, transparency about the deliberative process, stakeholder representation, conflict management, and the ethical rationale for contested limits becomes especially important (for details, see [14]). The point is not to allege impropriety, but to note that public, funder, and community confidence is better served when the justification pathway to prescribed actions, obligations, and standards is publicly visible.

## Conclusion

The 2025 update to the ISSCR guidelines is a significant and welcome advance. It moves human SCBEM oversight away from the previous integrated/non-integrated distinction, brings protocols leading to spatially organized 3D human SCBEMs within Category 2, and maintains categorical prohibitions on reproductive pathways. To secure consistent, ethically grounded implementation, we have proposed four targeted clarifications. First, a narrowly framed *consultation trigger* would help ensure that defined integration-building steps are brought to the attention of oversight committees when they are planned or undertaken, without expanding specialized review to ordinary organoid, endometrial, placental, trophoblast, or animal-model research. Second, Category 3B should distinguish permissible, time-limited implantation modelling from protocols aimed at sustained gestational or ectogenetic progression. Third, animal implantation and/or gestation findings should inform scientific context but should not be treated as *sufficient* or *decisive* grounds for loosening human SCBEM safeguards. Fourth, the *Guidelines* should reduce ambiguity by linking SCBEM classification to embryo-level organization or milestones in 3D and should make explicit the ethical rationale for its stated limits in preclinical human SCBEM research. These clarifications retain the architecture of the ISSCR framework while making it easier to apply, harder to misinterpret, and better justified in the ISSCR’s own terms.

## Data Availability

No datasets were generated or analysed during the current study.
